# Modeling therapeutic response to radioiodine in metastatic thyroid cancer: a proof-of-concept study for individualized medicine

**DOI:** 10.18632/oncotarget.16637

**Published:** 2017-03-29

**Authors:** Dominique Barbolosi, Ilyssa Summer, Christophe Meille, Raphaël Serre, Antony Kelly, Slimane Zerdoud, Claire Bournaud, Claire Schvartz, Michel Toubeau, Marie-Elisabeth Toubert, Isabelle Keller, David Taïeb

**Affiliations:** ^1^ SMARTc Pharmacokinetics Unit, Aix-Marseille Université, Inserm S911 CRO2, Marseille, France; ^2^ Service de Médecine Nucléaire, Centre Jean Perrin, 63011 Clermont-Ferrand Cedex 01, France; ^3^ Oncopole, 31059 Toulouse Cedex 9, France; ^4^ Hospices Civils de Lyon, Groupement Hospitalier Est, Service de Médecine Nucléaire, 69 677 Bron Cedex, France; ^5^ CLCC Institut Jean Godinot, 51056 REIMS CEDEX, France; ^6^ Service de Médecine Nucléaire, Centre Georges François Leclerc, 21079 Dijon Cedex, France; ^7^ Service de Médecine Nucléaire, Hôpital Saint-Louis APHP, 75475 Paris Cedex 10, France; ^8^ Service de Médecine Nucléaire, Hôpital Saint Antoine, APHP, 75012 Paris, France; ^9^ Service de Médecine Nucléaire, CHU La Timone, Aix-Marseille Université, 13385 Marseille 05, France

**Keywords:** metastatic thyroid cancer, radioactive iodine therapy, personalized medicine, mathematical model, therapeutic nuclear medicine

## Abstract

**Purpose:**

Radioiodine therapy (RAI) has traditionally been used as treatment for metastatic thyroid cancer, based on its ability to concentrate iodine. Propositions to maximize tumor response with minimizing toxicity, must recognize the infinite possibilities of empirical tests. Therefore, an approach of this study was to build a mathematical model describing tumor growth with the kinetics of thyroglobulin (Tg) concentrations over time, following RAI for metastatic thyroid cancer.

**Experimental Design:**

Data from 50 patients with metastatic papillary thyroid carcinoma treated within eight French institutions, followed over 3 years after initial RAI treatments, were included in the model. A semi-mechanistic mathematical model that describes the tumor growth under RAI treatment was designed.

**Results:**

Our model was able to separate patients who responded to RAI from those who did not, concordant with the physicians' determination of therapeutic response. The estimated tumor doubling-time ($T_{d}$ was found to be the most informative parameter for the distinction between responders and non-responders. The model was also able to reclassify particular patients in early treatment stages.

**Conclusions:**

The results of the model present classification criteria that could indicate whether patients will respond or not to RAI treatment, and provide the opportunity to perform personalized management plans.

## INTRODUCTION

Radioiodine therapy (RAI) has been used in the treatment of metastatic thyroid cancer since the 1940's [1]. This therapeutic radiation is based on the ability of well-differentiated papillary or follicular thyroid cancer cells to absorb and concentrate iodine. However, these cells show reduced expression of the sodium/iodide symporter (NIS) and thyroid peroxidase (TPO) compared to thyroid epithelial cells, which may account for the lower values of radioiodine uptake and effective period in thyroid cancer tissue. The efficacy of radioiodine therapy not only depends on the physiological targeting of the isotope treatment used in these studies, but also on the following additional factors:

1. The ability to up-regulate iodine transporter expression by increased Thyroid-Stimulating Hormone (TSH) levels, with either endogenous TSH or recombinant human TSH.
2. Optimal patient selection for RAI based on tumor characterization for NIS expression by molecular imaging.
3. The use of supra-physiologic thyroid hormone replacement to achieve TSH suppression between therapeutic cycles.

Iodine-avid metastases from thyroid cancer are generally sensitive to RAI and associated with excellent prognosis. Best responders to RAI are patients with highly avid metastases without structural correlate on high-resolution imaging studies, most frequently occurring in young subjects [2]. Often, distant metastatic lesions that are not ablated with ^131^I, may lose the ability to concentrate isotope ^131^I, possibly due to the clearance of well-differentiated tumor cells, and the persistence and expansion of less differentiated cells. Under these circumstances, ^131^I becomes ineffective and the treatment paradigm of progressive disease shifts toward a molecular-targeted therapy, which combines kinase inhibition and anti-angiogenic effects.

Currently, dosing regimens of RAI are typically selected based on clinical experiment and indicate tumor response in terms of serum thyroglobulin ($T_{g}$ levels, post^131^I therapy scintigraphy and anatomical imaging. However, treatment schedules that refer to administered 131-I activities and time interval between cycles are purely empirical.

Various dosimetric approaches have been proposed in order to achieve an optimal ratio of tumor to healthy tissues absorbed dose. Individual dosimetry provides a personalized therapeutic approach by maximizing the radiation absorbed dose to the tumor with preservation of at-risk organs [3]. From a radiobiological standpoint, however, there are many differences between internal and external radiotherapy. During radionuclide therapy, the cells and organs irradiated on, last not only for seconds or minutes, but also continuously over a long period of time, while the same absorbed dose is delivered. Therefore, the radiobiological model is complex and needs to be adjusted to radionuclide therapy. Beyond the model, dosimetry often requires prolonged, demanding procedures in order to collect measurements such as, blood and urine samples, acquisition of emission images at multiple time-points, and measurements of external dose rates [4]. Lastly, there are still unidentified individual susceptibilities to radiation. Furthermore, there is a crucial concern of potentially over/under dosing patients. Therefore, it is of major importance to develop alternative or complementary strategies.

The important approach is to determine which protocols would be the most effective to patients with minimal cumulative RAI activity in order to reduce potentially secondary side effects [5]. This ideal scenario is limited by the fact that empirical tests have infinite possibilities. A reasonable approach, however, is to construct a mathematical model capable of describing how the actions of the drug effects the characteristics of cancer and host. Once models are applied to actual, available population data, they must be validated by a variety of validation techniques. At that time, models could be granted the next level of investigation: simulating various in silico protocols of treatment administration. From this stage, the optimal outcome will surface, depending on the models development based on the project focus.

A key challenge that remains is interpreting the reality of the produced optimal scheme, such that the results should be in accordance with actual predications [6]. Currently, a rising effective strategy relies within “PK-PD” (pharmacokinetic-pharmacodynamic) models. As an example of proof of concept, we mention the phase I clinical trial named “Model 1” which was carried out in the framework of metastatic breast cancer and whose administration of the docetaxel-epirubicin doublet was entirely managed by a mathematical model [7, 8]. The results of this work allowed for the success in distributing the maximum tolerated doses over 14-day cycles, while respecting haematological toxicity constraints. Similarly, a mathematical model designed an ongoing Phase Ia/Ib clinical trial, for patients with metastatic NSCLC or malignant pleural mesothelioma. This model directed the oral administration of vinorelbine by metronomic schemes, which ensured optimal anti-angiogenic effects and acceptable haematological toxicities. Finally, more recently in Serre and al. a mathematical model was proposed to optimize the management of coupling RTX-Immunotherapy (Anti-PDL1, Anti-CTLA4) [9].

In metastatic thyroid cancer, serum thyroglobulin ($T_{g}$ levels are a major surrogate marker of responding to RAI. Hence, the aim of the work was to build a semi-mechanistic mathematical model that describes the tumor growth under RAI treatment using the thyroglobulin levels $\left(T_{g}\right)$. Note, that the main goal of the proposed model is not only to describe the time course of thyroglobulin, but rather the tumor doubling time (*Td*) of the disease under RAI treatment. We have chosen to draw as much information as possible from the Tg, the measure of which is the most reproducible with the minimum of bias between two different centers. Therefore, we voluntarily decided not to introduce imaging data into the model.

## RESULTS

### Patients' characteristics and outcomes

50 patients were enrolled in the study, where the ages ranged from 8 to 55 years with an average age of 29 years. Patients were initially treated by total thyroidectomy with lymph node dissection, with the exception of 3 cases, followed by RAI. pTNM stage was pT1/pT2 in 7 cases, pT3/T4 in 43 cases, N1/Nx in 47/3 respectively, and M1 in all cases. Patients were treated with a fixed RAI activity ranging from 3.7 to 5.5 GBq, apart from one case. The youngest patient of 8 years old, exceptionally, received 1.85 GBq for their first treatment. A fixed administration of RAI activity every 6-8 months was the most frequently prescribed schedule. The mean cumulative activity was 22.2 GBq. Eighty percent of the patients were clinically classified as responders with the following criteria (all present): decrease in stimulated Tg values over the time, decrease of tumor uptake or number of RAI-avid foci on post-therapy whole body scan (WBS), absence of newly diagnosed lesions on WBS, and absence of disease progression on regular radiologic evaluations.

### Performance of the prediction model

The parameters for our model were estimated using data from 50 patients treated for metastatic thyroid cancer.

For each patient throughout the treatment duration, empirical data for the concentration of stimulated Tg was available at each treatment cycle, prior to RAI administration. This data was used in the parameterization process for our model.

The population and inter-individual variability values for the model parameters were of the Nonlinear Mixed Effect Models (NLME) type. The six parameters of the final model, $\rho ,a,\lambda ,k_{e},T_{d},N_{0}$, were estimated using Monolix software (version 2016R1 Antony, France: Lixoft SAS, 2016). These parameters were assumed to be log-normally distributed amongst the individuals in the population, to insure biological relevant values. For the residual error, a normal distribution with proportional variance was chosen.

The first estimations showed that parameters ρ, a, λ had weak variability, with relative standard error (R.S.E) <2%. These parameters were subsequently fixed. Parameter $\;\beta_{T_{d}}$ proves the classification is statically significant with a p-value of 1.4e-005. The results obtained for the estimation of population parameters are summarized in Table 1.

**Table 1:** Estimations of Population Parameters

Parameter	Value	S.E. (S.A.)	R.S.E. (%)
**Population**			
$N_{z}$	1.12e+009	1.2e+003	0
$k_{e}$	0.319	0.068	21
*λ*	3.86e-009	-	-
*ρ*	0.00407	-	-
*a*	0.0169	-	-
$T_{d}$	9.8	1.6	16
$\beta_{T_{d}}$	1.92	0.44	23
**Interindividual Variability**			
$N_{z}$	2.05e-007	-	-
$k_{e}$	1.16	0.23	20
*λ*	2.47	0.34	14
*ρ*	0	-	-
*a*	0	-	-
$T_{d}$	0.3	-	-
**Mixture**			
b	0.372	0.031	8
$\pi_{\mathrm{cat}1}$	0.275	0.089	32
$\pi_{\mathrm{cat}2}$	0.725	0.089	12
**Group Parameters**			
$T_{d\;\mathrm{cat}=1}$	9.8	1.6	16
$T_{d\;\mathrm{cat}=2}$	66.6	29	44

Parameter estimates are displayed based of the estimation outputs from Monolix software. The values for the population parameters were found, along with their inter-individual variability. The bottom three values correspond to the confirmation of the mixture algorithm. Bottom: Mixture algorithm from Monolix used to estimate the mode.

### Classification of responders vs non-responders

**Figure 1:**
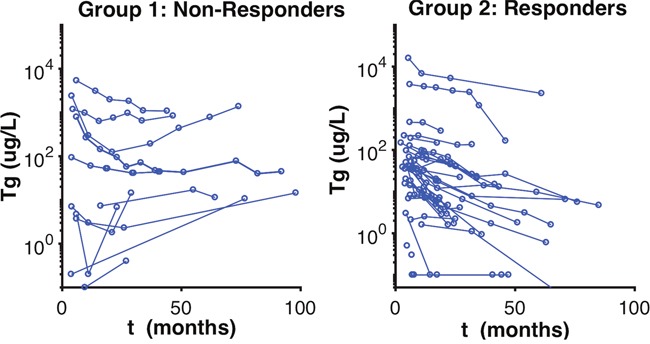
Observed Stimulated Tg Classification of Patients Observed stimulated Tg values of patients. This figure represents the group classification visualized by the mixture algorithm by the model, which separates the patients according to the pace of the thyroglobulin evolution curve.

The model distinguished two groups of patients. The kinetics of stimulated Tg values for both groups is represented in Figure 1. The variation of the tumor doubling time under treatment, represented by parameter $T_{d}$, illustrates the distinction within these patient groups as: responders vs. non-responders. $T_{d}$ was considered a key quantitative index of treatment effectiveness. The mixture procedure implemented in Monolix accurately separated patient groups: Group 1 (cat1) and Group 2 (cat2) for non-responders and responders, respectively. From Table 1, we have that 27.5% of patients were classified as non-responders and 72.5% as responders, from the mixture parameters $\pi_{1,2}$. These results are in accordance with the physicians' classification of 80% responders.

**Figure 2:**
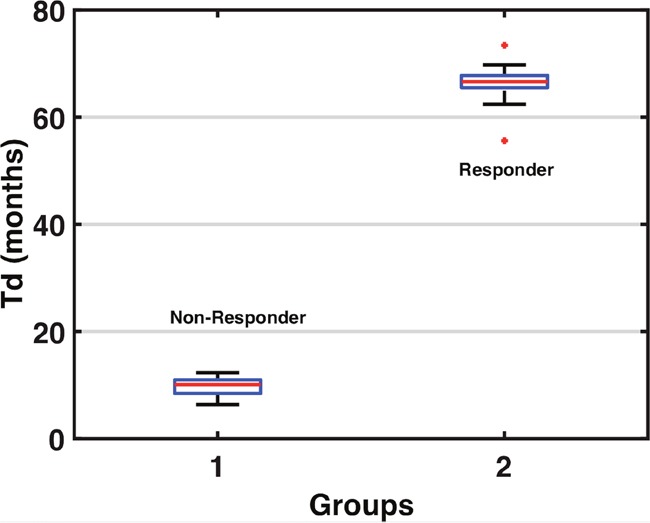
Tumor Doubling Time Group Classifications The boxplot above separates the groups via the tumor doubling, from non-responder Td average 9.8 months, and responder Td average 66.6 months.

**Figure 3:**
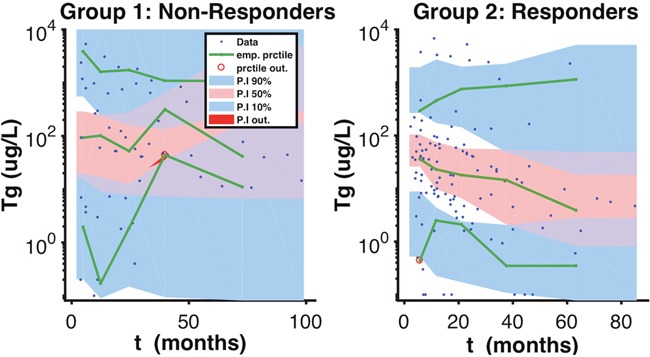
Visual Predicted Check Scheme A visual predicted check scheme was done that showed all empirical percentiles were within the corresponding 90% pharmacodynamic confidence intervals.

The non-responder group was classified by $T_{d}$ having a mean of 9.8 months, with a R.S.E of 16%, and responders were classified by $T_{d}$ having a mean of 66.6 months, with a R.S.E of 44% (Table 1). We can see a clear separation of groups by the Td in the boxplot (Figure 2). A visual check scheme was done that showed all empirical percentiles were within the corresponding 90% pharmacodynamic confidence intervals (Figure 3).

### Selected cases

**Figure 4:**
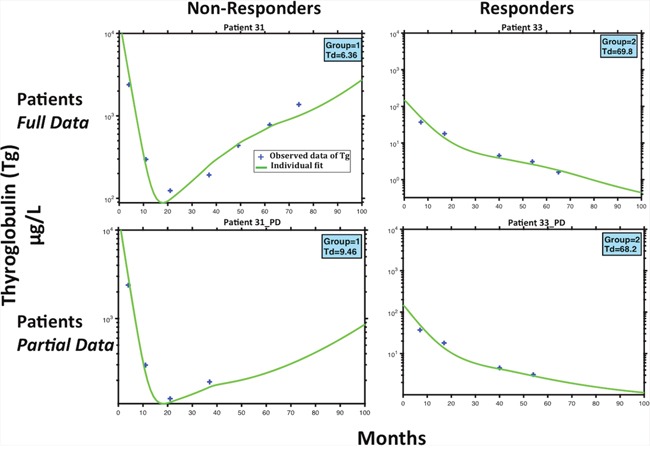
Patient Example Predictions Model fits for two patients classified as non-responder (P31) and responder (P33). The top 2 subfigures represent the full patient stimulated Tg data and model fits. The bottom two represents the same patients, while using less stimulated Tg data points. The importance of this figure is to demonstrate that while having limited data, the model can still classify patients to whither they will respond or not to treatment.

Figure 4 shows that with using only partial data for Tg, the model was still able to classify the patients accordingly. This dynamic is showed under Patient(s) 31,33 _(PD). Regarding patient 31, the model predicts their status as a non-responder depending on an estimation of their $T_{d}$, as 6.36 months, which is a relatively short doubling time in the setting of thyroid cancer. The status was also accurately predicted with the first 4 stimulated Tg-values ($T_{d}$ of 9.46 months) (Figure 4). Similarly, patient 33 was classified as a responder with estimation of the $T_{d}$ of 69.8 and 68.2 months, with respectively 5 or 4 stimulated Tg-values (Figure 4). These two patients provide evidence that the status could be predicted with the first stimulated-Tg values.

## DISCUSSION

In the recent years, mathematical modeling has gained an increased role for optimizing therapeutic strategies in oncology [6]. This present study shows mathematics can be used to model the Tg kinetics during RAI in metastatic thyroid cancer patients, and could provide perspectives for possibly applying modeling in the clinical decision making process.

This study suggests that it would be possible to distinguish the earliest possible responding patients by estimating the tumor doubling-time parameter, $T_{d}$, for each patient that follows 3 to 4 treatments. This work could be used as a tool to help guide clinicians to treat more efficient administration, by having the potential to minimize the administration dose and/or frequency of RAI iodine 131. The model was able to distinguish between responders or non-responders to RAI with agreement with the clinical classification based on Tg kinetics and physicians imaging. The proposed model takes into account several biologically relevant parameters and the effect of RAI, including the estimated effect. The model showed that the tumor doubling-time ($T_{d}$, was the most informative parameter for distinguishing responder from non-responders and could be used as a new classifier. From a mathematical standpoint, the parameters of this model were estimated with minimization algorithms, which makes it possible to find key parameters relative for a given patient, the best fit between the observed values of stimulated $T_{g}$, and the predicted curve calculated from the equations of the model. The mixture algorithm implemented in the software Monolix split the population into two classes: responders and non-responders to RAI. The p-value for mixture parameter $\beta_{T_{d}}$, $p=1.4\times 10^{-5}$, which proves the classification is statically significant. Classification was discordant for 2 patients between the models' classifier and the physician's treatment response assessment. Since, these patients exhibited a slight decrease in Tg without progression, they were classified by the physicians as responders.

From our example of selected patients, we show the model predicts the same classification as non-responders or responders, with full or partial data. Observing patient 31, the model predicts their classification as a non-responder, with only using the first five measurements of the observed stimulated $T_{g}$ from the initial eight, subsequently after 4 treatments of ^131^I an activity of 3.7 GBq. With only these first few measurements of $T_{g}$, the estimation of $T_{d}$ is 9.46 months, which although is a short amount of time, was found to be sufficient to classify Patient 31 in group 1 (Fig. 4, Patient 31_PD) as a non-responder. The same classification was accurate while saving three additional treatments. Similarly, using only the first 4 values of $T_{g}$, the estimation of the $T_{d}$ for patient 33 is 68.2 months, which is very large, but also sufficient to see that the patient 33 was in the responders group (Fig. 4, Patient 33_PD). The same classification was accurate while saving two additional treatments. We could deduce that these patients were over treated, however, this remains speculative in the absence of a randomization. Patient 31 was recognized as non-responders after the third treatment, but received three additional doses of 5.5 GBq over a period greater than 40 months, which were excessive, and perhaps could have been avoided if there would have been a criterion to characterize non-responders. Likewise, the model predicted that the $T_{g}$ for patient 33 was constantly decreasing after the fourth treatment, and was subsequently classified as a responder. However, this patient received an additional two treatments of 3.7 GBq, which could be unnecessary to achieve the same results. Moreover, we have investigated by stimulation, the option of increasing the activity from the start of treatment from 3.7 Gbq to 5.5 Gbq every four months over 80 months, in order to verify whether the patient would have responded to a higher density treatment over shorter cycles. However, our results show that Patient 31 remained as a non-responder.

In the recent years, several models have been proposed in oncology for improving drug delivery and efficacy [7–15]. We have also developed a similar approach in the evaluation of patients with high serum prostate specific antigen (PSA) levels with excellent preliminary results in the distinction between benign and malignant prostate lesions [16–18]. Our preliminary results open new perspectives for individualized management of patients. As this has already been done in certain phase I studies [7, 19], the model here could bring guidance for clinicians towards improving adaptive treatment, after identifying the parameters for each patient. The use of a model could help clinicians tailor strategies towards personalized medicine.

Our patient population was very specific with small RAI positive lung metastases, explaining the high rate of response to RAI [2]. The next step would be to validate our model in another independent population with similar disease. After validation, a clinical study could be designed: empirical vs model-assisted decision. After 3 cycles of RAI therapies, Tg levels could be inserted into the model. Based on the predicted tumor doubling time, the model will classify the patients with a certain percentage into a responder or non-responding group. The model could detect that the patient will not respond to RAI treatment. In this event, it could be suggested by the clinician to stop treatment, due to unnecessary secondary side effects, and potentially to find other more effective treatment options (TSH suppression alone, TKI). If the patient is classified to respond to RAI, the power of the model could suggest the duration response time, due to the delayed radioactive iodine effect which continues to act against cancer cells over time. In this event, the next treatment activity could be administered before the rise in Td is predicted. The advantage of this is to disperse the administration of RAI over time.

Our model allows for a quantitative interpretation of varying *Tg* concentration, according to the administered treatments. Using the model, the *Tg* concentration plays an intermediate role to provide an estimation of the *Td* doubling time, which was found as the key parameter representative of the evolution of tumor cells. The *Td* provides information to clinicians, which only partially reflects tumor behavior, and does not represent full accuracy to be used individually, but can be a tool to aid in the decision making process. The models classification of the tumor doubling time average estimates were 66.6 months and 9.8 months for responders and non-responders, respectively (p<10^−3^). The $T_{d}$ was found to decrease over time for responders, a result suggesting that RAI could change the behavior of a treated tumor. The mechanism is still unknown, but has also been observed with anticarcinoembryonic antigen pretargeted radioimmunotherapy in progressive medullary thyroid carcinoma [20]. Similarly, studies have quantitatively found that the ${\text{T}}_{\text{d}}$, was used as a parameter to monitor early response to treatment for brain cancer [21].

In clinical practice, when a patient is being monitored under treatment for various changes for thyroglobulin levels, clinicians are exposed to this data. Ideally, there are treatment decisions to be made at a given point during the treatment regimen. With limited data, it would be useful to have various criterions to assist with these decisions. We pointed out that the model classified patients with all the given data, and then with only the first few data points. The importance here is that if the model can still classify the responsiveness of patients early, then various treatment options could become more readily available; such as lowering or increasing RAI administrated activities, abolishing treatment, shifting to other approaches.

There are some additional parameters that might interplay and influence the reliability of the model. For example, the change in TSH can influence Tg levels, or the tumor size having small metastases <1mm, could be less sensitive to RAI, since most of the dose from beta particles are deposited outside these micrometastases [22]. However, these parameters would be difficult to realistically integrate into the model, but physicians could still consider its use and should maintain knowledge of the additional information that can be used in the interpretation of the model parameters. In some rare cases, stimulated Tg could be low despite the presence of distant metastases probably due to defects in Tg expression in tumor cells [23]. In this cases, the model can not be applied. The evaluation of post-therapy scan could help to identify these rare cases and prevent the application of the model.

Nevertheless, an extension of this work could also be envisioned for patients with refractory thyroid cancer, using more sophisticated mathematical models describing metastatic disease [11, 24] and joint administration of chemotherapy and TKI, in order to increase synergy effect and controlling toxicity [25]. Finally, we can also use the recent work to test whether one can derive a benefit from an abscopal effect in the synergy RTX and immunotherapy [9].

We can summarize our approach by acknowledging how mathematical modeling is a powerful tool, which provide novel insights from biology to physicians. In conclusion, this study shows that mathematical modeling accurately predicts Tg kinetics, which is foundational in the evaluation of RAI response. With the assistance from the model, the estimated tumor doubling time under RAI therapy (Td) can give us a direct understanding of disease, which could open new perspectives for computational benefits and collaboration in the clinical treatment decision making process.

## MATERIALS AND METHODS

### Study design

Clinical and biochemical characteristics, treatment regimens, and outcomes of metastatic thyroid cancer patients treated with RAI were **retrospectively collected** at eight referential institutions in France.

### Eligibility criteria

The inclusion criteria were: papillary thyroid carcinoma demonstrated from initial pathological report, presence of diffuse lung metastases at diagnosis on post-therapy scan, at least 3 courses of radioiodine (RAI) therapy during follow-up, endogenous TSH stimulation (i.e., hypothyroidism) for preparation to RAI, at least 3 years of follow-up after initial RAI ablation, pulmonary nodules <1 cm in diameter on CT, which are performed within the first 2 years post diagnosis.

Non-inclusion criteria were: presence of bone metastasis at diagnosis, other thyroid cancer histologic types or variants, and presence of anti-Tg antibodies at diagnosis.

### Mathematical model

**Figure 5:**
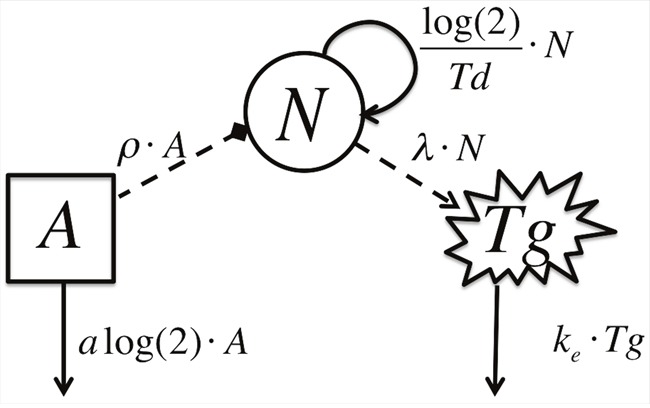
Schematic Model Diagram *A* represents the activity of radioiodine, *N*, represents the metastatic thyroid cancer cell count and *Tg*, represents the thyroglobulin concentration. Solid lines depict model flow between compartments. Dashed lines depict interactions between compartments.

Our model was constructed to focus on the interactions between radioiodine activity, metastatic thyroid cancer cells, and thyroglobulin concentration. The model encompasses three compartments. The radioactive iodine activity (RAI) is the input compartment *(A)*, which irradiates the tumor cells *(N)*. The output of the model is reflected by the concentration of the thyroglobulin level *(Tg)*. The given information used includes the administered quantity and frequency of the RAI activity and the post stimulated *Tg* levels, prior to each treatment administration. From these two given input and output sources, the model permits to deduce the unknown information for the tumor cell quantity (cells), which is representative of the disease state (Figure 5).

We let A(t) represent the RAI activity at time t and the constant a(inmonth−1), will describe the delayed target effect over time of the iodine on cancer cells, where the effect duration could be more than two times higher than the half-life of the iodine 131, which is 8 days, on average. If the activity $A_{0}$ (GBq) is administrated at on the onset of each treatment, we have

$$\frac{\text{dA}}{\text{dt}}\left(t\right)=-a\text{ Log}(2)A(t)$$

It is important to note that the above equation does not describe the physical half-life of RAI, but rather its effect on cancer cells acting with a continuous, low dose irradiation flow. This assumption allows RAI to continue to kill off cancer cells slowly over time. Numerous studies have proposed other sophisticated models to describe tumor growth [11]. For example, it is known that in absence of treatment, tumor growth can be well described by a Gompertz model, as reported in several studies [26]. Nevertheless, our observation time range is limited to 3-4 years, in which an exponential growth model provides a suitable approximation of tumor growth

To account for the effect of RAI on the tumor cells, the tumor growth can be modeled as:

$$\begin{array}{cc} \frac{\text{dN}}{\text{dt}}\left(t\right)=\frac{\mathrm{Log}\left(2\right)}{T_{d}}\;N\left(t\right)-\rho A(t)N(t) & N\left(0\right)=N_{0}, \end{array}$$

where $N_{0}$ is the initial size (in number of cells) of the tumor at the onset of treatment. As $N_{0}$ is not given in the data, it is unknown, and therefore estimated. The tumor doubling time under treatment, ${\text{T}}_{\text{d}}(\text{inmonth})$, is assumed to be constant during the treatment period. Following this idea, $T_{d}$ is not the natural tumor doubling time, but rather takes the role as the tumor doubling time under RAI treatment. The constant ρ(${\text{GB}}_{q}\times mo^{-1})$ becomes an efficiency parameter of the RAI effect on cancer cells.

We shall denote $T_{g}\left(t\right)$ as the concentration (μg/L)) of thyroglobulin at time t in the blood.

With drug administration, we set that:

$$\begin{array}{cc} \frac{dT_{g}}{\text{dt}}(t)=-k_{e}T_{g}+\lambda N\left(t\right) & T_{g}\left(0\right)=T_{g,\;0} \end{array}$$

where ${\text{k}}_{\text{e}}({\text{inmonth}}^{-1})$ is the constant elimination of thyroglobulin from the blood and the constant λ $(\mu g\times L^{-1}\times mo^{-1})\;$ denote the concentration of thyroglobulin per unit time produced by one cell, and $T_{g}(0)$ denotes the first value of $T_{g}$ prior to the first treatment.

This last equation represents the evolution of thyroglobulin concentrations according to the treatment administered. It is important to note that our accumulated patient data did not include measurements for the exact direct tumor mass volume, as could be assessed by anatomical imaging. Thus, the tumor mass was estimated, and treated as an unknown parameter, $N_{0}$. The model had estimated the tumor doubling time under treatment, Td, based on the previously measured stimulated thyroglobulin level (Tg) from data. Thus, $N_{0}$ was indirectly estimated based on Tg, via the interactions through Td.

Hence, the proposed model is managed by 6 parameters: $\rho ,a,\lambda ,k_{e},T_{d},N_{0}$ described in Table 2, along with the variables used.

**Table 2:** Description of Variables and Parameters of the model

Variable	Description	*Units*
*A*	Radioiodine Therapy Activity	$GB_{q}$
*N*	Tumor Cells	*cells*
$T_{g}$	Thyroglobulin Concentration	μg/L
Parameter		
*a*	Delayed target iodine effectiveness rate	1/mo
$T_{d}$	Tumor doubling time under treatment	mo
*ρ*	Efficiency rate of iodine on tumor cells	1/$GB_{q}$*× mo)*
$k_{e}$	Elimination rate of thyroglobulin from blood	1/mo
*λ*	Concentration of thyroglobulin produced by one tumor cell	μg/*(L× mo)*

The model is constructed with three variables, representing the interactions between RAI treatment, metastatic thyroid cells and thyroglobulin concentration. The five parameters of the model are described along with their corresponding units.
